# The Estimation of Pulse Tension

**Published:** 1907-03-23

**Authors:** George Parker

**Affiliations:** Physician to the General Hospital and Lecturer at University College, Bristol


### HOSPITAL CLINICS.

### THE ESTIMATION OF PULSE TENSION.

### A Clinical Lecture,

By GEORGE PARKER, M.A., M.D., Physician to the General Hospital and Lecturer at University College, Bristol.

I NEED not discuss the importance of obtaining a true measure of the tension of the blood, for we are all familiar with the many dangers which arise as this varies from the normal in one direction or the other. I would, however, remind you that not only are these dangers found under widely different conditions, such as shock, collapse, and syncope on the one hand, and cerebral hæmorrhage on the other, but also that alterations in the blood-pressure are matters of serious import in the course of most diseases. Clearly, then, to estimate this pressure correctly is one of the first things we have to do, whether from the view of prognosis or treatment.

The skilled finger by itself can give us valuable information, but unfortunately this is not always exact enough for our wants, nor is it absolutely reliable in every case. We may find ourselves in consequence sometimes doubtful, for example, whether we have to do with a cerebral hæmorrhage or a thrombosis. Or we may be unable to detect an ominous fall of pressure in pneumonia or the rise which indicates perforation in typhoid. Moreover, scientific tests show that some forms of the pulse as determined by the finger are apt to be misleading. Thus a small contracted vessel may give the idea of low pressure, because the finger gives us the total pressure of the tube instead of the pressure on a unit of surface. We must therefore employ all possible means of correcting our estimate, and must not neglect any available data as to the state of the circulation from other parts of the organism. Thus we should note the energy of the heart as governing the amount of the outflow of blood and the velocity of its expulsion. We should also mark the peripheral resistance and the elasticity of the vessels, since the tension is the result of two forces, the driving force of the heart and large vessels and the resistance opposed to this. Furthermore, we want to know not merely the absolute pressure, but whether it is what is normal and proper for the individual under the circumstances. Thus the normal pressure varies from infancy to middle life, and from that to old age, and there are other changes due to posture, food, and temperature, besides fluctuations according to the hour of the day. The tension which is normal in a vigorous middle-aged man would be excessive in a child or a weakly old woman. In all these matters we must correct and supplement the knowledge which is given us by the finger placed on the radial pulse. Above all, the sphygmometer in recent years has enabled us to get a more exact measure in many cases than was possible before. Its use shows us how fallacious our judgment has often been, and how frequently we have missed an important symptom. To educate our fingers as much as possible we cannot do better than to estimate first the tension with the finger, and then apply the sphygmometer and compare the results. After a short experience of this practice we shall find that we are far more competent to judge of a pulse, and that our finger sensations have a new meaning for us. Every student should go through such a course, and should not rest satisfied till he has compared his own estimate with that of a sphygmometer in some hundreds of cases, for though the instrument is easy to use, we cannot always have it with us, and in numberless instances we must rely upon our fingers alone.

Now in the pulse of a perfectly healthy individual we note that the vessel, which is flattened between the waves under gentle pressure, fills quickly and evenly, and after a brief interval collapses gradually, an almost imperceptible break, due to the dicrotic wave, occurring during the process of emptying. All this is repeated with regularity at a rate which is the normal for the individual. To judge how far a pulse departs from the standard great care should be taken in palpating it. The patient should not be excited and should be sitting or reclining, with his arm supported, but not by the hand which palpates the pulse. Some observers prefer to use one finger only, but Gibson advises that three fingers should be laid upon the artery, the index finger being nearest the patient's heart, so that the effects of pressure exercised by this finger may be observed through the other fingers. John Dacre recommends that the index fingers of both hands be used simultaneously, the one for observing the pressure required to obliterate the pulse and the other for registering the effect. The state of the vessel wall should be examined. If hard and resistant, rolling from side to side under pressure, we must take care not to confuse the cord-like feeling of the vessel with the tension of the pulse. In this condition of arterio-sclerosis the pressure may be normal, as in cases due to syphilis and diabetes; or it may be raised, as in many cases of kidney disease. Still less ought we to confuse high tension with calcified or atheromatous vessels. Indeed both these states are compatible with low pressure. The fullness of the vessel as judged by its size between the beats of the pulse, is no indication of the tension, and when the pulse itself is large and bounding the tension in a full artery is actually low.

It is often said that we measure the blood-pressure by the force necessary to obliterate the pulse. Gibson, however, lays down that it is the force needed to obliterate the artery in the interval between the pulsations. What is meant by obliteration of the artery in the interval? We want to exclude all reference to the force of the actual beats themselves, and to measure, under slight compression, the resistance remaining between the beats. This feeling of resistance, especially when the tension is high, is soon recognised by practice, though perhaps difficult to define. It must, of course, be distinguished from the sensation given by a thickened vessel which remains unchanged however great is the pressure which we employ. Whereas in a healthy vessel, when the pulse is just obliterated, a little additional pressure will flatten the artery completely.

If we take the other view, that the measure of the blood-pressure is the force needed to obliterate the pulse, we must clearly get very different results. What is the meaning of the difference? I think that in Gibson's method we are trying to get the diastolic pressure, that is the pressure remaining after the force of the systolic wave has been eliminated; and in the other we content ourselves with the total systolic pressure, noting as well as we can any great diastolic fall. Practically, as the finger gives only such a rough estimate, the results of the two methods are less discordant than would be anticipated. If you can, estimate the pressure by Gibson's method; but if you reckon by the obliteration of the pulse, you must allow for any special fall. Both the systolic and diastolic pressures are important, and can be separately measured by instruments of precision. The systolic is produced during the ventricular systole, and the diastolic is theoretically the pressure during the interval. The ratio between the two varies considerably, and is called the "pulse pressure"—a rather ambiguous term, by the way. If the driving force is high and the resistance low, the ratio is represented by a high figure, and *vice versa*. With a Riva-Rocci spyhygometer it is proved that the force needed to obliterate the pulse is just equal to the systolic pressure. Instruments such as Hill and Barnard's, which mark the point of greatest oscillation, show, at least approximately, the diastolic pressure, and it is possible also to read this on a Riva-Rocci with a little care. The systolic pressure depends chiefly on the force of the heart, while the diastolic is some indication of the peripheral resistance, though at no time is the pressure entirely dependent on a single factor. The sphygmograph gives an even more detailed analysis of the pulse, but its great defect is that it does not give a direct measure of the pressure.

To return to palpation by the finger, we have already alluded to the risk of mistaking the total pressure for that on the unit of surface, and the consequent under-estimation of the "small" pulse. Thus a contracted vessel under high tension, when even a strong cardiac systole has little dilating effect, may be mistaken for an empty vessel of low tension with a weak cardiac systole. In such a case it may not be at all easy to carry out Gibson's rule of obliterating the artery in the interval; but it should be aimed at.

In other pulses, again, we may find some peculiarity, such as marked dicrotism, which will guide us in our estimation; while in fat and œdematous patients it may be difficult to make out anything by the finger at all.

When we have done all we can to make our pulse palpation free from avoidable errors we look round for other corrections. First we should note, as Broadbent reminds us, the state of the heart, its size, the situation of the apex beat, the character of the impulse, the quality of the first sound at the apex, and of the second in the aortic and pulmonary areas. With a powerful impulse, clear first sound, and accentuated aortic second sound, the tension is usually high, unless the ventricle is receiving a deficient supply of blood. The deficiency may be due to valvular trouble, extensive lung disease, adherent pericardium, or pressure on the right ventricle from a distended abdomen; but whatever the source be, the heart in such a case will produce little effect by its work.

On the other hand, we may consider whether anything is causing vaso-contraction or the reverse. The direct signs of these states are not always available, but we know that pyrexia produces dilatation, and kidney disease very frequently vaso-contraction, and in some other conditions similar help may be obtained to enable us to build up a correct decision as to the blood-pressure. Again, supposing that the pulse tension is apparently high, and we learn that the patient is short of breath on exertion, that he complains of fullness and throbbing in the head, and tightness in the chest, if the cardiac symptoms are in accord, there can be little hesitation in deciding that excessive blood-tension is really present. If, on the other hand, the pulse appears unduly soft, we may find confirmatory evidence in the acceleration of its rate, in the feeble irregular action of the heart, or in the presence of fever, toxic conditions, surgical shock, syncope, or severe hæmorrhage. Much more might be said on the means of confirming the judgment we form through the finger. Unless we use all the side help we can get, even constant practice in feeling the pulse will not enable us to make accurate estimations, but with care wonderful precision can be obtained, more especially if we have trained ourselves by often testing our results by the sphygmometer.

